# Franz Diffusion Cell Approach for Pre-Formulation Characterisation of Ketoprofen Semi-Solid Dosage Forms

**DOI:** 10.3390/pharmaceutics10030148

**Published:** 2018-09-05

**Authors:** Constain H. Salamanca, Alvaro Barrera-Ocampo, Juan C. Lasso, Nathalia Camacho, Cristhian J. Yarce

**Affiliations:** Departamento de Ciencias Farmacéuticas, Facultad de Ciencias Naturales, Universidad Icesi, Calle 18 No. 122-135, Cali 760031, Colombia; chsalamanca@icesi.edu.co (C.H.S.); aabarrera@icesi.edu.co (A.B.-O.); jcamilolassor@gmail.com (J.C.L.); nathalia.camacho@correoicesi.edu.co (N.C.)

**Keywords:** ketoprofen, permeation assay, Franz cells, semi-solid systems

## Abstract

This study aimed to evaluate and compare, using the methodology of Franz diffusion cells, the ketoprofen (KTP) releasing profiles of two formulations: A gel and a conventional suspension. The second aim was to show that this methodology might be easily applied for the development of semi-solid prototypes and claim proof in pre-formulation stages. Drug release analysis was carried out under physiological conditions (pH: 5.6 to 7.4; ionic strength 0.15 M; at 37 °C) for 24 h. Three independent vertical Franz cells were used with a nominal volume of the acceptor compartment of 125 mL and a diffusion area of 2.5 cm^2^. Additionally, two different membranes were evaluated: A generic type (regenerated cellulose) and a transdermal simulation type (Strat-M^®^). The KTP permeation profiles demonstrated that depending on the membrane type and the vehicle used, the permeation is strongly affected. High permeation efficiencies were obtained for the gel formulation, and the opposite effect was observed for the suspension formulation. Moreover, the permeation studies using Strat-M membranes represent a reproducible methodology, which is easy to implement for pre-formulation stage or performance evaluation of semi-solid pharmaceutical products for topical or transdermal administration.

## 1. Introduction

With the remarkable interest in the development of innovative pharmaceutical products worldwide, suitable methods need to be established to evaluate the fundamental physicochemical parameters required in pre-formulation studies. Formulation studies are essential for drug effectiveness reliability, both in approved drugs and new pharmaceutical formulations. Simple methods must be implemented for determining the main physicochemical parameters including solubility, partition coefficient and dissolution profiles. In the case of topical formulations, for which the drug is released through the skin, evaluation of the permeation is critical to establish bioavailability and thereby make an approximation of the effectiveness. Additionally, for semi-solid dosage forms aimed for topical or transdermal application, methodologies to test and verify the performance in product pre-formulation stages are essential to avoid additional performance tests once the product is market-ready [1,2,3,4].

Franz Cells are a widely used methodology to evaluate in vitro drug permeation [3,5,6], which have advantages, such as (i) few handling of tissues, (ii) no continuous sample collecting and (iii) low amount of drug required for analysis. With the rise of personalised medicine, it is necessary to develop various pharmaceutical dosage forms for the same active molecule allowing the variability of administration and dosage. Nonsteroidal Anti-Inflammatory Drugs (NSAIDs) cover a range of indications and would require various dosages, formulations and administration forms as they are used for chronic as well as acute conditions such as in the treatment of chronic joint diseases (i.e., osteoarthritis and rheumatoid arthritis) and inflammatory diseases (i.e., acute fractures, sprains and sports injuries) [7,8,9]. Some NSAIDs commonly used in medical practice include acetylsalicylic acid, ketoprofen (KTP), ibuprofen, naproxen, indomethacin and piroxicam [9,10,11,12,13,14]. These drugs carry out their pharmacological action by decreasing inflammation, fever and pain processes.

Despite the therapeutic benefits, these drugs have many adverse effects that affect gastrointestinal, renal and cardiovascular systems and the liver due to the unspecific blocking of the enzyme cyclooxygenase 1. Most NSAIDs are well absorbed when administered orally and are widely distributed throughout the body [13,15]. In contrast, NSAID topical administration has a localised effect and a reduced risk of systemic adverse effects [14,16]. This dosage form also avoids hepatic first-pass metabolism, allowing for sustained drug release, and is easy to apply and remove in the case of side effects [17].

KTP is an NSAID widely used in the treatment of rheumatoid arthritis, osteoarthritis, ankylosing spondylitis and acute episodes of gout [14]. This drug is often formulated as a gel for topical application. Technically, the skin represents a limitation to the use these formulations because the stratum corneum (outermost layer) provides not only a barrier against absorption of chemical and biological toxins but also hinders drug penetration [18]. Because of the potential that the formulations of NSAIDs for topical use represent for the treatment of inflammatory chronic and acute conditions, we evaluate the permeation rate of a gel of KTP in semi-solid state using Franz cells simulating specific conditions of human skin. Using this methodology, we aimed to establish the physicochemical parameters needed to predict the therapeutic effect of topical dosage forms.

## 2. Material and Methods

### 2.1. Materials

The reagents and materials used for this study were: Methylene blue (MB), HCl, NaOH, KH_2_PO_4_, K_2_HPO_4_, KCl, calcium chloride and sodium alginate provided by Merck (Burlington, MA, USA). KTP as active pharmaceutical ingredient (API) was donated by Sanofi-Genfar-Laboratories (Cali, Colombia), and it was used as received. Ultra-pure water (pH 5.5 and 1 μS/cm conductivity) was obtained from the purification system Ellix Essential Millipore^®^ (Darmstadt, Germany). Cellulose membranes for dialysis, which retain most proteins of 12 kDa or greater, were acquired from Sigma-Aldrich (St. Louis, MO, USA), and Strat-M^TM^ membranes were purchased from Millipore (Temecula, MA, USA). The Strat-M^TM^ comprises two layers of polyethersulfone on top of one layer of polyolefin. These polymeric layers create a porous structure with a gradient across the membrane in terms of pore size and diffusivity. The porous structure is impregnated with a proprietary blend of synthetic lipids, imparting additional skin-like properties to the synthetic membrane.

### 2.2. Preparation of Buffer Solutions 

Buffer solutions of pH 5.6 and 7.4 were prepared using HCl/NaOH and KH_2_PO_4_/K_2_HPO_4_, respectively. Ionic strength was adjusted to 0.15 M using KCl according to methodologies described in United States Pharmacopoeia [19].

### 2.3. Preparation of Ketoprofen (KTP) Semi-Solid Formulations

The preparation of KTP suspension was performed using 12.5 g of KTP, 2 mL of glycerine and 2.5 g of sodium alginate as suspensor ingredient and 500 mL of ultra-pure water. For this, KTP was mixed with glycerine until forming a “paste”, which was added in water and stirred for 2 h at 30 °C. Then, the sodium alginate was gradually added with slow stirring until forming a hetero-dispersed system. On the other hand, the development of KTP gel was carried out using 12.5 g of KTP, 2 mL of glycerine, 2.5 g of sodium alginate, calcium chloride salt and 500 mL of ultra-pure water. First, the KTP suspension was prepared as mentioned above. Then, the calcium salt was gradually added with slow stirring until forming a gel via the cross-linking between the polymer chains and the salt. In both formulations, the KTP concentration was 2.5% *w*/*w*. The characterisation of KTP suspension and gel was carried out at 37 °C, using a pH-meter with a conductivity meter (Mettler Toledo^®^ S213, Columbus, OH, USA). The viscosity was measured by a viscometer (Brookfield DV-IPrime, Middleboro, MA, USA) coupled to needle #1 at 100 rpm, whereas the zeta potential measurements were performed using a Zetasizer Nano ZSP (Malvern Instruments, Cambridge, UK) with a disposable folded capillary cell (DTS1070). Each measure was carried out in triplicate and prepared as fresh formulation prior the permeation assay.

### 2.4. Methods

#### 2.4.1. Determination of KTP Permeation

The experiments were conducted in three independent vertical Franz cells (A, B and C) with a nominal volume of the acceptor compartment of 125 mL and a diffusion area of 2.5 cm^2^. For the donor compartment, an amount of 5 g of each formulation (2.5% *w*/*w* KTP) was initially set. The experiments were conducted in triplicate, carried out at 37 °C and 480 rpm for 24 h. Samples were evaluated at different time points, and data analysis was made by comparing the releasing efficiency (PE) values.

The assays were performed in two aqueous media as acceptor phases mimicking physiological conditions corresponding to buffer solutions at pH 5.6 and pH 7.4 at 0.15 M. Also, two types of membranes were used: A regenerated cellulose membrane with molecular weight cut-off at 12 kDa and a transdermal diffusion test model Strat-M^®^ [20].

Releasing efficiency was defined in terms of the mass flux (*J*) [21,22], which describes the change of drug permeation with respect to time in aqueous systems. In our study, the mass flux (mol·cm^−2^·h^−1^) was determined using the AUC of the permeation profile recorded at a specific time interval and is related to the rectangular area (*R*) described by 100% of the permeation process at the same time interval (24 h). 

Mass flux can be calculated from:

(1) $$Flux\;\left(J\right)=\frac{\int_{0}^{t}y\;dt}{y_{100}t}\times 100\%$$

where *y*_100_ is the AUC value assumed with a permeation of 100% in a time interval *t*, and *y* represents the AUC value of the permeated drug during the same time interval. 

#### 2.4.2. Quantification of KTP

The quantification of KTP was performed using a spectrophotometer at a wavelength of 259 nm coupled to a CPS temperature control system at 37 °C (Shimadzu, Columbia, MD, USA). For measuring, 1 mL of each sample was taken from the acceptor compartment during different time points, and a similar volume of the aqueous media was added to retain the sink conditions in the system by keeping a constant initial volume.

#### 2.4.3. Kinetic Study of the KTP Permeation

To investigate the kinetics and mechanisms governing KTP permeation through membranes, the following kinetic models were applied:

(i) The zero-order model: This model is widely used for pharmaceutical dosage systems that do not disintegrate and have a very slow drug release. Furthermore, this model assumes that the area of the tablet does not change significantly and material balance conditions are not formed. This model is expressed by the equation:
  (2) $$Q_{t}=Q_{0}+k_{0}t$$
  where *Q_t_* is the amount of dissolved drug at time *t*, *Q*_0_ is the initial amount of drug in the solution (most of cases *Q*_0_ = 0) and *k*_0_ corresponds to the constant of zero-order release [23].
(ii) The first-order model: This model is commonly used to describe the absorption and release of water soluble drugs from porous matrices. This model can be expressed by the equation:
  (3) $$LogQ_{t}=LogQ_{0}-\frac{k_{1}}{2.303}t$$
  where *Q_t_* is the amount of dissolved drug at time *t*, *Q*_0_ is the initial amount of drug in the solution and *k*_1_ corresponds to the constant of first-order release [24].
(iii) The Higuchi model: This model is widely used to describe the release of soluble and sparingly soluble drugs in aqueous media, from various semi-solid and/or solid matrices according to the equation:
  (4) $$Q_{t}=k_{H}t^{1/2}$$
  where *k_H_* is the Higuchi dissolution constant, whereas *Q_t_* and *t* correspond to the parameters described previously [25,26].
(iv) The Korsmeyer-Peppas model: This is a generalised model of the Higuchi equation that allows one to explain drug delivery mechanisms where erosion and/or dissolution of the matrix occurs. This model has been widely used to describe the drug release from polymer systems. The related equation is:
  (5) $$\frac{M_{t}}{M_{\infty }}=k_{r}t^{n}$$
  where *M_t_*/*M*_∞_ corresponds to the fraction of drug released at time *t*; *k_r_* is the release constant which is characteristic for the polymer–drug interactions, whereas *n* is the diffusion exponent that is characteristic for the release mechanism. When *n* equals 0.5, the equation becomes equal to the Higuchi model, indicating that the release mechanism is of a Fickian type (case I), whereas values of n between 0.5 and 1.0 suggest that the release mechanism corresponds to an anomalous (non-Fickian) transport. Values of 1.0 indicate that the release mechanism is similar to a zero-order release, whereas values of n greater than 1.0 (Super Case II transport) suggest a drug release process dependent on the relaxation of the polymer chains in the matrix, passing from a vitreous state (lower kinetic movement and increased potential energy) to a relaxed state rubber type (high kinetic movement and lower potential energy) [22,27].

### 2.5. Data Processing and Analysis

Data were tabulated and analysed using Microsoft Excel (Redmond, WA, USA) and GraphPad Prism 7 (La Jolla, CA, USA), respectively. Variance homogeneity of the data was evaluated using the Bartlett test. The values of permeation efficiency were compared by one-way ANOVA. To determine the differences between independent groups, Tukey’s *post-hoc* test was applied. A confidence level of 95% was adopted, and data are presented as mean ± standard deviation.

## 3. Results and Discussion 

### 3.1. Quantification of KTP

Results show that the methodology adopted to determine the KTP concentration had a high level of repeatability as demonstrated by coefficients of variation below 5%. Also, the values of the linear regression analysis revealed that the methodology fits the linear model of Beer-Lambert (Table 1). 

### 3.2. Characterisation of KTP Matrix

Table 2 shows the results of the physicochemical characterisation performed on the two formulations.

These results show that the gel formulation has a higher electrical stability due to a higher zeta potential value (~−33.4 mV). In the gel formulation, both the charge direction and the viscosity are created by the cross-linking of sodium alginate as a polymeric material and calcium chloride salt forming a very structured and stable vehicle. In the suspension formulation, while the polymeric material was the same, no calcium salt was used, thus viscosity was strongly reduced. Similar results have been obtained for conductivity measures, where the gel formulation has a higher conductivity in the presence of the CaCl_2_ electrolyte.

### 3.3. Determination of KTP Permeation

**Figure 1:**
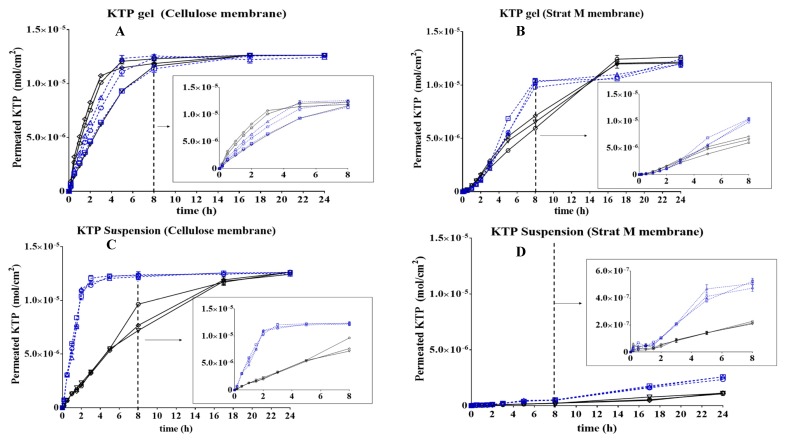
Permeation profiles of ketoprofen (KTP) for 24 h in different conditions of matrix, medium, pH and type of membrane. (**A**) KTP Gel-cellulose membrane, (**B**) KTP Gel—Strat M^®^ membrane, (**C**) KTP Suspension—cellulose membrane, (**D**) KTP Suspension—Strat M^®^ membrane. Graph legends: **◯** = Cell A. pH: 5.6; **☐** = Cell B. pH: 5.6; **△** = Cell C. pH: 5.6; ▽ = Cell A. pH: 7.4; ◇ = Cell B. pH: 7.4; 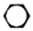 = Cell C. pH: 7.4.

Figure 1 shows the results of the in vitro permeation profiles of KTP using three independent Franz cells for 24 h with the 8 h behaviour shown in a highlighted graph. 

The permeation profiles show a marked dependence on the type of dosage matrix used, as well as on the media pH and the membrane type used in the Franz cell. With cellulose membranes (Figure 1A), the concentration of the drug is changing as a function of time, until a plateau is reached at 7 h from the experiment start. It is important to note that there is no apparent difference in the amount of KTP released between both media pH, primarily due to the cellulose membrane pH stability and secondarily due to the drug reservoir property of gel matrix conferring KTP a protection against ionisation processes mediated by pH [28]. In the experiment with Stat-M membrane presented in Figure 1B, although the membrane is not cellulose, the drug release behaviour is very close to what is observed in Figure 1A, implying that the permeation profile of KTP results from the type of matrix used. Two different permeation profiles of KTP gel were obtained using Strat-M membranes (Figure 1B), showing that drug permeation depends on the physiological medium. At pH 5.6, two relevant changes were observed. (i) A fast permeation process occurred during the first 8 h reaching ~1.0 × 10^−5^ mol/cm^2^. Then, (ii) a decrease in the drug release profile keeping the amount of KTP around a constant value until 24 h.

When the KTP suspension permeation was evaluated in cellulose membranes (Figure 1C), a similar behaviour as the KTP in gel formulation was observed. At pH 5.6, the permeation was fast reaching a maximum value of ~1.0 × 10^−5^ mol/cm^2^ after 3 h, whereas at pH 7.4, the permeation was stepwise reaching the maximum concentration level after 24 h. At pH 7.4, the permeation capacity is lower when the molecule has carried out an ionisation process compared with the almost neutral form of KTP at pH 5.6. (the pKa value for KTP is 4.54). Most significantly, the type of matrix is important in the case of the suspension dosage form, which does not have the same protective structure against pH as the gel formulation. Thus, the suspension is not an encapsulation vehicle for KTP and drug permeation is dependent on the molecule’s physicochemical properties as the pH change of the acceptor medium in the Franz cell affected the most KTP permeation rate.

When suspensions were evaluated in Strat-M membranes (Figure 1D), permeation was very low under pH values of 5.6 and 7.4, reaching ~6.0 × 10^−7^ mol/cm^2^ as the maximum permeated amount. First of all, given the capacity of Strat-M membranes to simulate human skin, the permeation rates are slower than the rates observed with cellulose membranes. Strat-M has two different layers simulating human epidermis and dermis, thus for the drug to permeate through, it needs to overcome two different structures, which takes more time in the Franz cell experiment. Also, the suspension formulation has not shown a protective effect against pH, and the drug permeation rate is dependent on the matrix.

Briefly, these results show that the Franz cell experiment can be applied in the comparison of two semi-solid dosage forms for KTP at the pre-formulation stage as proof of concept of the membrane permeation claim in topical formulations. Determination of the mass flux and the lag time in both gel and suspension represent important evaluation parameters. The mass flux is determined as the area under the curve in Figure 1 and is expressed in mol·cm^−2^·h^−1^, whereas the lag time is expressed in hour. These are summarised in Table 3.

The mass flux (*J*) values for the KTP gel were lower with the Strat-M membrane than with the cellulose membrane, and the effect of the pH on the permeation profile of the drug is more marked at pH 7.4, likely due to ionisation of KTP as mentioned above. Additionally, when KTP is in suspension, the type of membrane affects significantly the permeation, allowing for the discrimination between the effect of the medium and the type of vehicle used. Indeed, information on the effect of membranes is very common when analysed in conjunction with the drug type and matrix media [29]. In our study, the lowest values of flux are obtained using the Strat-M membrane at pH 7.4. This is a very interesting result for the qualification of semi-solid dosage forms in pre-formulation stages since it is possible to predict the behaviour of topical dosage forms.

Importantly, gel formulation allows for faster KTP release from the matrix at the beginning due to the lower latency period in comparison with suspension. The latency or lag time is a very useful parameter in Franz cell studies when comparing the performance of formulations or matrices for the same chemical compound [30]. While the cellulose membrane does not affect the diffusion of KTP from donor compartment to receptor compartment in Franz cells, Strat-M does so [31]. To provide an explanation for these results and relate them to a possible release mechanism, several semi-empirical kinetic models were evaluated.

### 3.4. Kinetic Study of the KTP Permeation

For this study, the zero-order, first-order, Higuchi and Korsmeyer-Peppas models were taken into account. The results obtained in the evaluation of these kinetic models are summarised in Table 4.

The results from the kinetic evaluation support the observations made in the prior sections. It is worth noting that the KTP permeation for the gel formulation shows a better adjustment to the Korsmeyer-Peppas model, where KTP releasing could be mediated by an erosion process of the gel matrix [32,33,34]. These observations are independent of the pH evaluated and are common when drugs are carried on a matrix vehicle. On the other hand, the KTP suspension with a slow permeation process is fitting mostly with the zero-order model showing that KTP permeation depends upon the availability of the drug and is related with the ionisation process. Therefore, KTP suspension is not an adequate vehicle as the pH in the environment will have an effect on the drug.

## 4. Conclusions

Permeation studies using Franz cells represent a highly reproducible methodology, which is easy to implement in any laboratory. In this model, the selection of the membrane is a critical step because generic membranes such as regenerated cellulose can provide evidence on important changes associated with the size of the drug evaluated and the vehicle used to dissolve it. Specialised membranes for transdermal simulation as Strat-M also provide important information on the permeation changes associated with the aforementioned variables. Regarding the permeation process of KTP, it was established that this parameter decreases slightly when the pH of the medium changes from 5.6 to 7.4 due to the degree of ionisation originated by the pKa of the drug. The kinetic evaluation is a good complement to the Franz diffusion experiment as it addresses the mechanism of releasing when the drug is interacting with a vehicle matrix. Finally, we demonstrate that the Franz cells model using cellulose and Strat-M membranes as a characterisation tool can be easily implemented for pre-development stage or during the evaluation of the performance of semi-solid pharmaceutical products for topical or transdermal administration.

## Figures and Tables

**Table 1:** Parameters evaluated during the standardisation of the methodology to quantify levels of ketoprofen (KTP) through UV-Visible spectrometry.

Medium	Linearity	
	Linear Equations	*R* ^2^
Buffer pH 7.4	*y* = 0.0623*x* − 0.0016	*R*^2^ = 0.9999
Buffer pH 5.6	*y* = 0.0635*x* + 0.0038	*R*^2^ = 0.9999

**Table 2:** Values of Z-potential, conductivity, pH and viscosity obtained for the two KTP formulations at 37 °C.

Matrix of KTP	Zeta Potential (mV)	Conductivity (µS/cm)	pH	Viscosity (Cp)
Gel	−33.4	962.6	6.8	35033
Simple Suspensión	−20.2	465.6	6.8	36.4

**Table 3:** AUC values for mass flux and lag time obtained from the KTP permeation profiles of the gel and the suspension using three cells and two membranes (cellulose and Strat-M membranes) in two media at 37 °C.

Matrix of KTP	Permeation Parameters	Conditions					
		Media pH: 5.6			Media pH: 7.4		
		Cell A	Cell B	Cell C	Cell A	Cell B	Cell C
Gel		Cellulose membrane					
	Flux (mol/cm^2^ h) × 10^4^	2.68 ± 0.08	2.53 ± 00.9	2.71 ± 0.01	2.54 ± 0.03	2.75 ± 0.08	2.77 ± 0.01
	Lag-time (h)	0.04	0.013	0.007	0.12	0.107	0.119
		Strat-M membrane					
	Flux (mol/cm^2^ h) × 10^4^	2.07 ± 0.08	2.12 ± 0.08	2.10 ± 0.08	1.96 ± 0.03	2.00 ± 0.09	1.94 ± 0.01
	Lag-time (h)	0.012	0.056	0.055	0.433	0.457	0.179
Simple suspension		Cellulose membrane					
	Flux (mol/cm^2^ h) × 10^4^	2.82 ± 0.04	2.83 ± 0.10	2.81 ± 0.07	2.03 ± 0.12	2.07 ± 0.05	2.18 ± 0.07
	Lag-time (h)	0.108	0.101	0.109	0.126	0.130	0.142
		Strat-M membrane					
	Flux (mol/cm^2^ h) × 10^4^	0.25 ± 0.03	0.27 ± 0.01	0.27 ± 0.01	0.18 ± 0.01	0.10 ± 0.02	0.10 ± 0.03
	Lag-time (h)	0.298	0.221	0.225	0.324	0.329	0.382

**Table 4:** Kinetic study for KTP permeation through cellulose and Strat-M^®^ membranes.

Formulation	pH of the Medium	Membrane	Zero Order		First Order		Higuchi		Korsmeyer-Peppas		
			*k*_0_	*R* ^2^	*k*_1_	*R* ^2^	*k_H_*	*R* ^2^	*k_r_*	N	*R* ^2^
Gel	5.6	Celullose	7.55 × 10^−9^	0.648	7.55 × 10^−11^	0.647	4.01 × 10^−7^	0.886	2.02 × 10^−7^	0.640	0.894
		Strat-M	9.13 × 10^−9^	0.799	9.13 × 10^−11^	0.835	4.08 × 10^−7^	0.897	1.24 × 10^−9^	1.380	0.945
	7.4	Celullose	9.40 × 10^−9^	0.607	9.40 × 10^−11^	0.607	4.01 × 10^−7^	0.784	3.40 × 10^−7^	0.565	0.864
		Strat-M	9.47 × 10^−9^	0.929	9.47 × 10^−11^	0.929	4.09 × 10^−7^	0.971	5.86 × 10^−9^	1.110	0.976
Suspension	5.6	Celullose	5.51 × 10^−9^	0.450	5.50 × 10^−11^	0.450	4.05 × 10^−7^	0.748	5.11 × 10^−7^	0.52	0.745
		Strat-M	1.75 × 10^−9^	0.993	1.74 × 10^−11^	0.991	7.06 × 10^−8^	0.910	1.39 × 10^−9^	0.981	0.928
	7.4	Celullose	9.73 × 10^−9^	0.908	9.73 × 10^−11^	0.907	3.56 × 10^−7^	0.973	3.48 × 10^−8^	0.857	0.978
		Strat-M	7.17 × 10^−10^	0.971	7.17 × 10^−12^	0.972	2.86 × 10^−8^	0.866	1.57 × 10^−9^	0.814	0.854
